# Undirected Sucrose Efflux Mitigation by the FT-Like SP6A Preferentially Enhances Tuber Resource Partitioning

**DOI:** 10.3389/fpls.2022.817909

**Published:** 2022-05-09

**Authors:** Bas van den Herik, Kirsten ten Tusscher

**Affiliations:** Computational Developmental Biology, Utrecht University, Utrecht, Netherlands

**Keywords:** resource partitioning, sucrose, phloem, xylem, SP6A, potato, biophysical model, SWEET

## Abstract

The yield of harvestable plant organs depends on overall photosynthetic output and the subsequent distribution of the produced assimilates from source leaves across different sink organs. In this study, we aimed to obtain, using a two-sink transport model, mechanistic understanding of how the interplay between sink and pathway properties together determines sink resource partitioning. As a working example, we analyzed the partitioning of resources within potato plants, investigating the determinants of tuber sink yield. Our results indicated that, contrary to earlier studies, with a spatially explicit biophysically detailed model, transport pathway properties significantly affect sink resource partitioning within the physiologically relevant domain. Additionally, we uncovered that xylem flow, through its hydraulic coupling to the phloem, and sucrose efflux along the phloem, also significantly affected resource partitioning. For tubers, it is the cumulative disadvantage compared to sink leaves (distance, xylem flow, and sucrose efflux) that enable an undirected SP6A-mediated reduction of sucrose efflux to preferentially benefit tuber resource partitioning. Combined with the SP6A-mediated sink strength increase, undirected SP6A introduction significantly enhances tuber resource partitioning.

## Introduction

Photosynthesis and the subsequent distribution of produced assimilates across different sink organs together determine the yield of harvestable plant organs. Most study efforts have focused on optimization of photosynthesis (Nölke et al., 2014; Driever et al., 2017). However, competition for sucrose between sink organs also has a major impact on final crop yield. An important question is, thus, which factors affect sucrose partitioning between sinks, how the impact of these factors depends on sucrose availability and environmental conditions, and how these different factors interact. As an example, in potato, upon tuber formation, sucrose delivery to tubers is substantially increased at the cost of other plant organs (Fernie et al., 2020). It is generally accepted that this enhanced tuber sucrose partitioning is achieved through a switch from symplastic to apoplastic unloading, increasing the sucrose unloading rate at tubers (Viola et al., 2001). However, this enhanced unloading operates against a background in which tuber organs may have a different affinity for sucrose than, e.g., plant roots, reside at a larger distance from source leaves than young developing sink leaves, and differ physiologically from sink leaves, which evaporate rather than take up water. To fully understand how much tuber unloading must increase for efficient tuber filling to occur and how this may be enhanced through targeted breeding, the importance of, and interplay with, other sink and transport pathway properties must be investigated.

Mathematical models have developed as an invaluable tool in investigating plant yield. However, to gain insight into factors determining resource partitioning, appropriate models considering all the relevant aspects are needed. Currently, in large-scale agronomic models aimed at predicting field level yields as a function of plant type and environmental conditions, sucrose partitioning between sinks is based on experimentally measured, developmental stage specific, partitioning tables (de Wit et al., 2019). As such, these models provide no insight in the mechanistic basis of sucrose partitioning between plant organs. More detailed models describing individual plant performance as a function of plant physiology and architecture, such as the frequently used functional–structural plant (FSP) models, typically assume that relative sink strength determines sucrose partitioning (Lescourret et al., 2011; Da Silva et al., 2014; de Vries et al., 2021). These latter models, thus, implicitly assume that properties of the transport pathway such as resistance, length, or relative nearness of different sinks to the source organs do not significantly impact sink resource allocation, which at least under certain conditions have been shown to be incorrect (Pallas et al., 2010).

In contrast, based on the generally accepted Münch hypothesis, biophysically detailed transport models describe sucrose and water transport as a convective flow resulting from an osmotically driven pressure gradient (Thompson and Holbrook, 2003; Hölttä et al., 2006; Lacointe and Minchin, 2008). Besides automatically integrating pathway resistance and length, more detailed biophysical models can also include the effects of xylem water flow (Hölttä et al., 2006, 2009) and radial sucrose efflux (Minchin and Lacointe, 2017; van den Herik et al., 2021). In a single-sink context, these biophysical transport models have highlighted the importance of pathway properties on transport dynamics, efficiency, and, thus, eventually sucrose delivery to sinks. However, currently available two-sink models have produced conflicting outcomes on the relevance of pathway properties for sucrose partitioning. Minchin et al. (1993) demonstrated that, due to pathway resistance effects, for two sinks differing in sink strength (v_max_), the sink with the lowest v_max_ obtained a larger fraction of available sucrose than expected based on the v_max_ ratios alone. In other words, the weaker sink receives more sugar relative to its v_max_ than the stronger sink, while in absolute numbers, the stronger sink is still dominant. This suggested that sink characteristics are not the sole determinants of resource competition. Paradoxically, results of a later study slightly extending the model of Minchin et al. (1993) by Bancal and Soltani (2002) suggested that for physiologically relevant source concentrations, this phenomenon is negligible. This would imply that within the relevant range, sink characteristics fully dictate resource partitioning. Importantly, both the models used a simplified phenomenological description of pathway resistance and its effect on transport, rather than explicitly modeling the osmotically driven pressure gradient driving transport. A recent study, integrating biophysically detailed transport dynamics in a multisink FSP model, instead indicated that both the distance and sink strength determine sugar partitioning in grape (Zhu et al., 2021). Thus, it remains unclear what the exact relevance of pathway properties is on resource partitioning and how this may depend on sink properties. A first goal of this study is, therefore, to investigate the impact of modeling choices for the relevance of pathway properties on resource partitioning.

While most often only the length and/or resistance of the pathway between source and sink is considered relevant for resource partitioning, *in planta* extensive radial water and sucrose transport occurs along this pathway as well, also potentially affecting resource distribution. In addition to their roles in source and sink loading and unloading (Braun et al., 2014), SWEET transporters also facilitate bidirectional, gradient-dependent sucrose transport between the phloem and the apoplast in the long-distance phloem, with sucrose export from the phloem dominating (Chen et al., 2012). Similarly, SUC/SUTs localized in the source region facilitate active, proton-coupled, sucrose loading into the phloem, while along the long-distance phloem, these SUC/SUTs facilitate retrieval of sucrose from the apoplastic space (Hafke et al., 2005). Apart from these transporters’ dependence on local sucrose levels, additional regulation of transport capacity takes place. As an example, in potato, it was recently shown that the phloem mobile peptide StSP6A, or so-called tuberigen, inducing the transition from stolon to tuber under short-day conditions, binds to and thereby reduces StSWEET11 transport capacity by approximately 40% (Abelenda et al., 2019). The relevance of sucrose efflux from the long-distance transport phloem on resource partitioning has, thusfar not been investigated. Additionally, while sink strength and affinity have been generally considered as important properties for resource partitioning, so far physiological sink properties, particularly the direction and rate of water exchange with the environment and, hence, xylem flow, have not been considered. A second goal of this study is, thus, to obtain a mechanistic understanding of how the interplay between sink and pathway properties (length, xylem flow, and sucrose efflux) together determines sink resource partitioning. As a working example, we will analyze the partitioning of resources within potato plants, investigating the determinants of tuber sink yield. The differences in sink strengths, locations, and physiological properties between developing leaves, roots, and tubers provide an interesting context for studying sink resource partitioning. To this end, we used our previously developed biophysical transport model parameterized for potato (van den Herik et al., 2021), which we here extended from a single sink to a two-sink model.

## Materials and Methods

According to the Münch hypothesis, plant sucrose transport in the phloem occurs through convective flow generated by an osmotically driven pressure gradient that needs to overcome pathway resistance. Depending on the study, researchers have made different assumptions on the relevance of pathway resistance, the significance of feedback effects of sucrose concentration (*via* sap viscosity) thereon and the importance of explicitly modeling water transport, and the associated radial and xylem water transport. Later, we briefly introduce three models for sucrose transport differing in these assumptions. To resolve whether the previously obtained paradoxical result that pathway resistance does not significantly impacts sink resource competition depends on modeling details, these models were compared with regard to their results on sucrose partitioning between two sinks:


(1) $$P\,C_{1}=\frac{v_{1}}{v_{1}+v_{2}}$$

where PC_1_ is the partitioning coefficient for sink 1, v_1_ and v_2_ are the respective unloading rates in the two sinks. For a system without radial solute flux, v_1_ + v_2_ is equal to the loading rate (v_0_).

### Model Overview and Underlying Assumptions

#### Model 1: No Resistance

**FIGURE 1:**
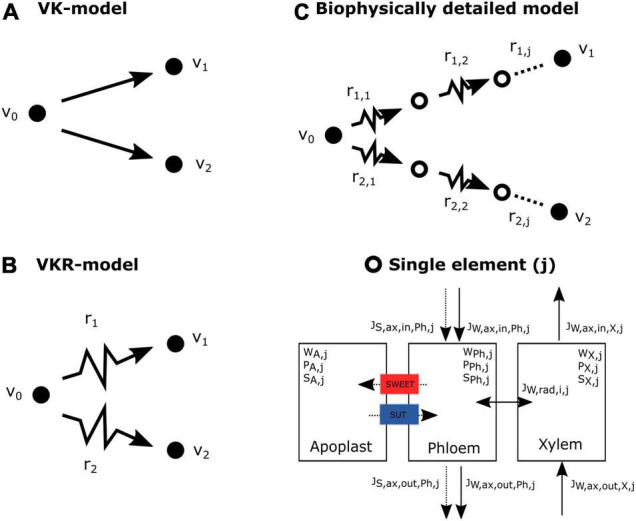
Model overview of the three compared models. **(A)** Resource partitioning in the VK model solely depends on the sink properties, as visualized by the straight lines leading from source (v_0_) to sinks. **(B)** Partitioning in the VKR model does not only depend on sink properties, but also depend on pathway resistance (r). **(C)** In the biophysically detailed model, pathway resistance depends on local conditions (open circles representing a single element). In a single element, water and sucrose flows are calculated based on local resistance, pressure, and sucrose concentration. Furthermore, a single element consists not only of a phloem compartment, but also contains a xylem and apoplastic compartment with which water, respectively, sucrose exchange occur.


The no resistance or VK model (sink strength, v_max_; sink affinity, k_m_) is the simplest model we introduce (Figure 1A). It assumes that there is no resistance in the phloem and, thus, no pressure gradient is required. Source concentration (c_0_) is equal to sink concentration (c_1_, c_2_) as a result. Sink unloading rates are modeled using the Michaelis–Menten equation, implying an active unloading mode. Differences in partitioning between the two sinks, thus, solely depend on differences in sink v_max_ and K_m_, enabling a simple analytical solution:

(2a) $$v\,1=v_{m\,a\,x,1}\,\frac{c_{1}}{c_{1}+k_{1}}$$

(2b) $$v\,2=v_{m\,a\,x,2}\,\frac{c_{2}}{c_{2}+k_{2}}$$

(2c) $$v_{0}=v_{1}+v_{2}$$

#### Model 2: Sucrose-Dependent Resistance

In the resistance or VKR model (Figure 1B), transport resistance is included by introducing a total pathway resistance term (r_*i*_) (Minchin et al., 1993). Consequently, source and sink concentrations are different (c_0_ ≠ c_1_ ≠ c_2_) and resistance can impact resource partitioning. Bancal and Soltani (2002) further extended this model by including the influence of sucrose concentration on pathway resistance *via* sap viscosity. Specifically, sucrose concentrations halfway the pathway (S_1/2_) are taken to calculate overall pathway resistance. The equation used to calculate sucrose-dependent resistance used by Bancal and Soltani (2002), valid for sucrose concentration between 0 and 1.5 M, is:


$$R_{s}=R_{3}(0.685S_{\frac{1}{2}}^{4}-1.0411S_{\frac{1}{2}}^{3}+0.9512S_{\frac{1}{2}}^{2}+0.1364S_{\frac{1}{2}}$$

(3) +0.3396)

where R_3_ is the reference viscosity at a sucrose concentration of 1 M. The equation above directly gives a relationship between sucrose concentration and pathway resistance. The equation is based on the viscosity effects on resistance, thus by replacing the reference resistance (R_3_) for a reference viscosity, the effect of sucrose concentration on viscosity is obtained.

For steady-state conditions, the unloading rates in the sinks can be found by numerically solving the system of equations below (i = 1, 2 for a 2-sink system):


(4) $$v_{0}-v_{i}=\frac{c\,0\,\left(c\,0-c_{i}\right)}{r_{i}}-\frac{v_{m\,a\,x,i}\,c_{i}}{k_{i}+c_{i}}=0$$

In the comparison here, we use this extended model of Bancal and Soltani (2002), including the sucrose-dependent pathway resistance.

#### Model 3: Spatially Explicit Sucrose and Water Flows

This biophysically detailed model explicitly describes water and solute flow in the phloem resulting from an osmotically driven pressure gradient as described by the Münch hypothesis (van den Herik et al., 2021; Figure 1C). Local pathway resistance depends on local phloem characteristics and sap viscosity (and, hence, sucrose concentrations), rather than a global pathway resistance being superimposed from calculated average pathway characteristics and sucrose concentrations. We initially only incorporated water (J_W, ax, Ph/X_) and sucrose phloem flow (J_S, ax, Ph_) as well as radial water flow (J_W, rad, Ph/X_) to ensure conservation of water mass (W) and sucrose (S). These radial fluxes were modeled using the following equation:


(5a) $$J_{W,r\,a\,d,P\,h,j}=L_{r}\,A_{r\,a\,d,j}\,\left(0-\Psi_{P\,h,j}\right),w\,i\,t\,h\,\Psi_{P\,h,j}=P_{P\,h,j}+\Pi_{P\,h,j}$$

where L_r_ is the radial hydraulic membrane permeability, A_rad,j_ is the radial area between phloem and xylem, Ψ_Ph_ is the phloem water potential, P_Ph_ is the phloem turgor pressure, and Π_Ph_ is the phloem osmotic potential. For later simulations, we also incorporated water flow in the xylem, which we described following the cohesion-tension theory. In these simulations, radial water exchange occurs between phloem and xylem, following similar approaches by Hölttä et al. (2006) and applied earlier by van den Herik et al. (2021):


(5b) $$J_{W,r\,a\,d,P\,h,j}=L_{r}\,A_{r\,a\,d,j}\,\left(\Psi_{X,j}-\Psi_{P\,h,j}\right),w\,i\,t\,h\,\Psi_{P\,h,j}=P_{P\,h,j}+\Pi_{P\,h,j}$$

Because of the complexity, the biophysically detailed model is solved numerically in an explicit geometry consisting of interconnected elements. For this study, we extended our previously developed model (van den Herik et al., 2021) to multiple sinks by endowing individual elements with the possibility for multiple inflow and outflow terms. Radial inflow and outflow can still be described by a single term within an element. Combined, this results in the reformulated water and sucrose mass balances below:


$$\frac{\partial W_{P\,h/X,j}}{\partial t}=(\sum_{i=1}^{n}J_{W,a\,x,i\,n,P\,h/X,j,i}-\sum_{i=1}^{m}J_{W,a\,x,o\,u\,t,P\,h/X,j,i}$$

(6a) $$+J_{W,r\,a\,d,P\,h/X,j})\rho$$

(6b) $$\frac{\partial S_{P\,h,j}}{\partial t}=\sum_{i=1}^{n}J_{S,a\,x,i\,n,P\,h,j,i}-\sum_{i=1}^{m}J_{S,a\,x,o\,u\,t,P\,h,j,i}+J_{S,R\,a\,d,P\,h,j}$$

where *n* is the amount of upward oriented elements connected to element j and m is the amount of downward oriented elements connected to element j. Descriptions of the variables and parameters are given in Tables 1, 2. A more detailed description of the model, its underlying assumptions, and potato-specific parameterization can be found in a study by van den Herik et al. (2021). While the original single-sink model incorporated a passive unloading mechanism, in this study, we included active unloading through incorporating the Michaelis–Menten equation to enable a one-to-one comparison with the VK and VKR model. Importantly, when comparing different types of sink organs—roots, leaves, and tubers—we maintain for all the sinks an active unloading mode. We do this despite stolon ends upon tuberization switching from an active to passive unloading mode (Viola et al., 2001). The reason for not incorporating a passive unloading-specific equation for tuber sinks is that this would introduce an additional dependence on the sucrose concentration assumed for tubers, complicating a comparison of how differences in sink properties such as xylem water transport and distance from source leaves affects resource partitioning.

**TABLE 1:** Symbols and units for the used variables/equations in the biophysically detailed model.

Variable	Symbol	Units
Water mass	W_Ph/X,j_	g
Sucrose	S_Ph,j_	mol
Pressure	P_Ph/X,j_	MPa
Sucrose concentration	c_Ph,j_	mol/m^3^
Axial water flow	J_W,ax,in/out,Ph/X,j_	g/s
Axial sucrose flow	J_S,ax,in/out,Ph/X,j_	mol/s
Radial water flow	J_W,rad,Ph/X,j_	g/s
Radial sucrose flow	J_S,rad,Ph,j_	mol/s
Water potential	_Ph/X,j_	MPa
Axial area	A_Ph/X,j_	m^2^
Radial area	A_rad,j_	m^2^
Osmotic potential phloem	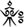 _Π,j_	MPa
Dynamic viscosity phloem	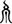 _μ,j_	MPa s
		

**TABLE 2:** Relevant parameters for the biophysically detailed model used in this study, for a full description of the model, parameters, and parameter estimation (see van den Herik et al. (2021)).

Parameter	Symbol	Value	Units
Density of water	ρ	0.998e6	g/m^3^
Radial hydraulic membrane permeability	L_r_	5e-8	m/MPa/s
Dynamic viscosity water/xylem	μ_x_	1.0019e-9	MPa s
Phloem sieve element radius	a_Ph_	8.4e-6 at t = 0	m
Xylem conduit radius	a_x_	30e-6	m
Axial permeability phloem	k_p_	3.82e-12	m^2^
Elastic modulus phloem	ε_p_	30	MPa
Elastic modulus xylem	ε_x_	750	MPa
Transpiration rate	J_trans_	0.02	g/s
Water potential soil	ψ_soil_	0	MPa

In this more detailed model, there is no single parameter (r) for total pathway resistance. Instead, local pathway resistance depends on local viscosity and pressure differences. To see this more clearly, we rewrote the phloem-specific water flow rate:


(7a) $$J_{w,a\,x,i\,n,P\,h}=A_{P\,h}\,\frac{k_{P\,h}}{\mu_{P\,h}}\,\frac{\Delta \,P_{P\,h}}{L}=N_{P\,h}\,\pi \,a_{P\,h}^{2}\,\frac{k_{P\,h}}{\mu_{P\,h}}\,\frac{\Delta \,P_{P\,h}}{L}$$

in terms of pressure differential and resistance (r):


(7b) $$J_{w,a\,x,i\,n,P\,h}=\frac{\Delta \,P_{P\,h}}{r_{P\,h}},w\,h\,e\,r\,e\,r_{P\,h}=\frac{\mu_{P\,h}\,L}{k_{P\,h}\,N_{P\,h}\,\pi \,a_{P\,h}^{2}}$$

This yielded an expression for pathway resistance, which is proportional to viscosity (μ) and pathway length (_L_) and inversely proportional to phloem axial permeability (k), number of phloem conduits (N), and individual phloem conduit radius (a). To study the effect of resistance on sucrose transport in this model and compare its effects to that in other models, we varied pathway length, as this parameter is directly proportional to resistance. While for the between-model comparison, we could have varied other resistance parameters from Eq. 7b instead, we decided to vary length as this also enabled us to investigate the effect of different source-sink distances on resource partitioning, assuming all other pathway properties are equal. Importantly, *in planta*, hydraulic architecture/axial permeability of phloem may vary over the length of the plant and/or between plant organs (Clerx et al., 2020), making the linear scaling of pathway length with resistance a simplifying assumption.

In the biophysical model, we used the equation for sucrose-dependent viscosity as described by Morison (2002), i.e., using the volume fraction of sucrose (Φ) to calculate the viscosity:


(8) $$\mu_{P\,h,j}=\mu_{X}\,e\,x\,p\,\left(\frac{4.68*0.956\,\phi_{P\,h,j}}{1-0.956\,\phi_{P\,h,j}}\right)\,w\,i\,t\,h\,\phi_{P\,h,j}=\frac{V_{s\,u\,c}\,S_{P\,h,j}}{V_{s\,u\,c}\,S_{P\,h,j}+V_{P\,h,j}}$$

Given that the VKR and biophysical model use different equations to calculate viscosity, we checked to what extent that this result in different sucrose concentrations to viscosity mapping, as this could potentially underlie differences in outcomes between the two models. Supplementary Figure 1 illustrates that despite the different applied equations, a highly similar mapping occurs, indicating that this cannot be the cause of differences in model results.

### Simulation Details and Model Implementation

Standard sink strength was set at v_max_ = 12.5 nmol/s, sink affinity was set at K_m_ = 75 mM, and pathway resistance was set at *r* = 7.5e12 Tmol s/m^6^ (for the VKR model) or pathway length was set at l = 0.25 m (for the biophysically detailed model). The pathway length of 0.25 m corresponds to an equal resistance, as the VKR model for a sugar concentration of 0 mM. That is, the resistances are equal for conditions in which viscosity effects of solutes are ignored. To investigate differences in resource partitioning between the three models, we considered three different scenarios in a single-source, two-sink system, with the two sinks differing in either sink strength (v_max_), sink affinity (K_m_), or pathway resistance (r). For the v_max_ scenario, we used 22.5 nmol/s for sink 1 and 2.5 nmol/s for sink 2; for the K_m_ scenario, we used 75 mM for sink 1 and 750 mM for sink 2; and for the resistance scenario, we used 7.5 and 150 Tmol s m^–6^ for the VKR model and 0.25 and 5 m for the biophysically detailed model. To compare the three models, steady-state solutions for a range of loading rates (0.025–25 nmol/s) were calculated and phloem-only conditions were used (Eq. 5a). Aforementioned values were taken from a study by Bancal and Soltani (2002), to enable comparison between their earlier results and our model outcomes. To validate the robustness of the analysis, we compared our results for a default zero resistance shared pathway to a non-zero resistance shared pathway from source to sink. This did not significantly affect the results (Supplementary Figure 2).

The impact of xylem flow on resource partitioning was investigated using a constant water uptake of sink 2 (2e–8 m^3^/s, equal to total uptake in a study by van den Herik et al., 2021) representing a root organ, while varying water flow in sink 1 between -1e-8 m^3^/s (water evaporation equal to source) and 2e–8 m^3^/s (water uptake equal to sink 2), representing organs on the continuum from leaf to tuber to root. Evaporation in the source region was set equal to the net sum of the water flow in both the sinks and simulations were performed for three different source loading rates. We also investigated 3 biologically realistic 2 sink scenarios (root-root, tuber-root, and young sink leaf-root), where we imposed a soil water potential of 0 MPa (Perämäki et al., 2001), simulating tubers by decreasing water uptake permeability by 90% relative to roots and young leaves through an evaporation rate of 10% of that of mature leaves.

Radial sucrose transport between phloem and apoplast and sucrose removal from the apoplast were modeled as done in a study by van den Herik et al. (2021). In summary, bidirectional SWEET transport facilitates efflux from the phloem (v_max_ = 0.148 mol/m^3^/s, K_m_ = 70 mM, Chen et al., 2012) and retrieval from the apoplast (v_max_ = 0.148 mol/m^3^/s, K_m_ = 10 mM, Chen et al., 2012), while SUT transporters (v_max_ = 0.117 mol/m^3^/s, K_m_ = 1 mM, Schulze et al., 2000) also facilitate retrieval from the apoplast to the phloem. Efflux and retrieval rates were implemented as mol per m^3^ per s and were, therefore, constant on a per length basis during the steady-state conditions at which we evaluate model outcomes. SP6A-mediated efflux mitigation was modeled by decreasing the v_max_ of SWEETs by 40%, as reported in a study by Abelenda et al. (2019).

The VK model was analytically solved. The system of equations describing the VKR model was solved in MATLAB R2020b using the build in function “fsolve” with a function threshold of 1e–13. Residual function values were checked to ensure the correctness of the solution and various initial values were used to search for alternative solutions of the system. We only considered biologically valid solutions, i.e., positive solute concentrations. The system of differential equations describing the biophysically detailed model was implemented in MATLAB R2020b and was solved using the build-in solver “ode15s,” using an integration time step of Δt = 1 s using a non-homogeneous mesh for loading (20 elements, 0.005 m/element), long-distance pathway (30 elements, 0.0083 m/element), and unloading zones (20 elements, 0.005 m/element), as described in detail by van den Herik et al. (2021). The source code for the biophysically detailed model and implementation of the VKR and VK models are available on https://tbb.bio.uu.nl/khwjtuss/TwoSinkSucroseTransport/.

## Results

To investigate whether the previously found limited importance of pathway resistance on sink resource partitioning depends on modeling details, we compared the previously used, simpler VKR model with the more detailed biophysical model, with the VK model serving as a baseline. First, we started with investigating model differences in a single-sink setting.

### Structural Underestimation of Sucrose Concentration and Resistance in the VKR Model

**FIGURE 2:**
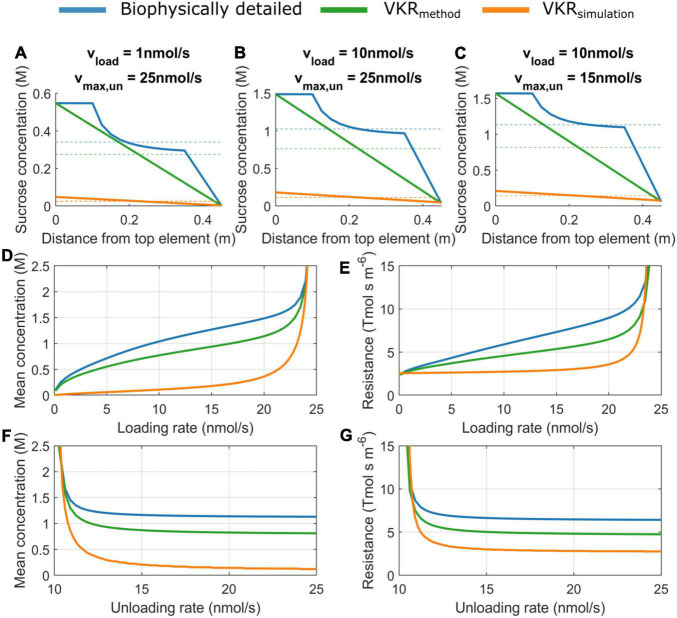
Sucrose concentration and resistance are underestimated when not including a spatial sucrose gradient. Concentration gradients for **(A)** v_load_ = 1 nmol/s, v_max,un_ = 25 nmol/s, **(B)** v_load_ = 10 nmol/s, v_max,un_ = 25 nmol/s **(C)**, v_load_ = 10 nmol/s, v_max,un_ = 15 nmol/s. Dotted lines indicate mean pathway sucrose concentration. **(D)** Mean concentration as a function of source loading rate for a constant unloading rate of 25 nmol/s and **(E)** mean pathway resistance as a function of source loading rate. **(F)** Mean concentration as a function of sink unloading rate for constant loading rate of 10 nmol/s and **(G)** mean pathway resistance as a function of sink unloading rate.

A major difference between the VKR and biophysical model is the manner in which sucrose phloem concentrations are used to calculate resistance. In the biophysical model, sucrose gradients are calculated in a spatially resolved manner with explicit source loading zones, sink unloading zones, and lateral water transport resulting in non-linear gradients (Figures 2A–C, blue lines). In contrast, the VKR model assumes a linear source-sink concentration gradient (Figures 2A–C, green lines indicate the linear gradient for the biophysical model). In the biophysical model, local pathway resistance is subsequently calculated from local sucrose concentration, while in the VKR model, an average, overall pathway resistance is calculated from mean pathway sucrose concentration. This results in an underestimation of pathway sucrose concentration (Figures 2D,F) and resistance (Figures 2E,G) when using a linear VKR-type gradient. The level of underestimation increases with concentration gradient steepness, i.e., increasing loading (Figure 2A vs. 2B and Figures 2D,E) and unloading rates (Figure 2B vs. 2C and Figures 2F,G).

Instead of extracting a VKR-type gradient from the biophysical model, we next explicitly simulate the VKR model using equal parameterization as for the biophysical model. Due to the dynamic feedback between resistance and concentration, the general tendency for lower resistances in the VKR model results in significantly less steep source-sink concentration gradients and lower source concentrations (Figures 2A–C, orange lines). Importantly, these lower source concentrations in the VKR model result in an overestimation of the minimum loading rates necessary to obtain physiologically relevant source concentrations (0.1–2 M).

### Effects of Resistance and Biophysical Detail on Resource Allocation

Next, we investigated the impact of using either a baseline VK, simple VKR, or the biophysical model on resource partitioning between two sinks. We hypothesized that the structural underestimation of pathway resistance in the VKR compared with the biophysically detailed model impacts resource partitioning.

### Sink Strength (V_max_)

**FIGURE 3:**
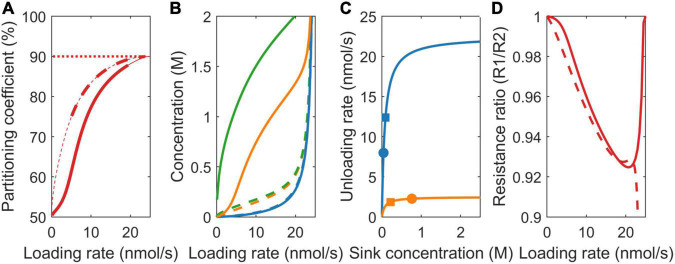
Impact of model choice on partitioning coefficients and the underlying mechanisms for different sink strengths (v_max_). **(A)** Partitioning coefficient for the VK (dotted), VKR (dashed), and biophysical (solid) models. The bold lines in **(A)** represent physiologically relevant conditions (0.1 < c_0_ < 2 M) showing that the biophysical model has a much broader physiologically relevant range. **(B)** Sink 1 (blue), sink 2 (orange), and source (green) sucrose concentrations. **(C)** The Michaelis–Menten curves for unloading rates, with dots representing the biophysical model and squares representing the VKR model. **(D)** Resistance ratios for the VKR (dashed) and biophysical (solid) models.

At the onset of tuberization, a switch from symplastic to apoplastic unloading mode increases tuber sucrose unloading rate. As a logical first scenario, we, thus, investigated the effect of differences in sink strength on resource partitioning. For v_max,1_ = 22.5 nmol/s and v_max,2_ = 2.5 nmol/s, the simple VK model predicts the partitioning coefficient to equal the v_max_ ratio (v_max,1_/v_max,2_ = 90%) (Figure 3A, dotted line). In absence of resistance, source concentration equals sink concentration, resulting in equal sink concentrations. This causes the sinks to be equally saturated, resulting in v_max_ values being the sole source of differences in sink uptake. In contrast, the VKR model predicts the partitioning coefficient to start at 50% and increase in a saturating manner toward the v_max_ ratio defined 90% (Figure 3A, dashed line). In the presence of resistance, differences between source and sink concentrations arise, allowing for between sink concentration differences. The higher unloading rate at sink 1 results in a lower local sink concentration (Figure 3B, dashed blue vs. dashed orange lines) and reduced saturation (Figure 3C, compare position of blue vs. orange squares on the saturation curves). This causes sink 1 resource partitioning to be lower than expected based on v_max_ ratio alone. Partitioning reaches the expected v_max_ defined value when both the sinks approach saturation at high loading rates (approximately 20 nmol/s).

The biophysically detailed model predicts a slower, sigmoidal increase to 90% partitioning (Figure 3A, solid line). In both the VKR and biophysical model, the higher v_max,1_ resulted in a lower c_1_, lower mean pathway concentration, and resistance, causing r_1_/r_2_ < 1 (Figure 3D). However, a lower c_1_ also results in a steeper concentration gradient c_0_-c_1_ that is more underestimated in the VKR model than the c_0_-c_2_ gradient. As a consequence, the VKR model overestimates r_1_-r_2_ differences, resulting in a r_1_/r_2_ ratio closer to 1 in the biophysical model (Figure 3D). Thus, while absolute resistance levels are higher in both the pathways, relative resistance differences between the two pathways are smaller in the biophysical model. Since the resistance difference acts against the v_max_ driven concentration gradient difference (lower resistance causes higher sink concentrations), the reduced relative resistance difference in the biophysical model allows for larger difference between sink concentration (Figure 3B) and saturation differences (Figure 3C, compare location of the dots representing the biophysical model with the squares representing the VKR model). This further diminishes the advantage of sink 1 from its higher v_max_. Summarizing, over a large range of loading rates, an increased unloading at the tubers (with higher v_max_) has less effect than expected based on unloading rates only.

### Sink Affinity (k_m_)

**FIGURE 4:**
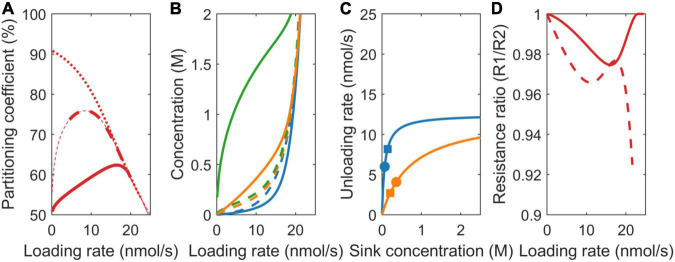
Impact of model choice on partitioning coefficients and the underlying mechanisms for different sink affinities (K_*m*_). **(A)** Partitioning coefficient for the VK (dotted), VKR (dashed), and biophysical (solid) models. The bold lines in **(A)** represent physiologically relevant conditions (0.1 < c_0_ < 2 M), showing that the biophysical model has a much broader physiologically relevant range. **(B)** Sink 1 (blue), sink 2 (orange), and source (green) sucrose concentrations. **(C)** The Michaelis–Menten curves for unloading rates, with dots representing the biophysical model and squares representing the VKR model. **(D)** Resistance ratios for the VKR (dashed) and biophysical (solid) models.

In addition to sink strength, plant sink organs may also differ in sucrose affinity. For higher sink 1 affinity (k_1_ = 75 mM, k_2_ = 750 mM), at equal sink concentrations, the effective uptake rate of sink 1 (v_1_) is significantly higher than that of sink 2 (v_2_). In the VK model, with its equal sink concentrations, this causes the partitioning coefficient to heavily favor sink 1 at low loading rates and, hence, sink concentrations (Figure 4A, dotted line). At higher loading rates, both the sinks saturate, causing the partitioning to converge to the 50% defined by the equal v_max_ values. In the VKR model, the higher affinity of sink 1 and, hence, higher uptake at low loading rates result in lower local concentration at sink 1 (Figure 4B). Consequently, even though k_2_ >> k_1_, sink 2 is more saturated than sink 1 (Figure 4C). As a result, partitioning to sink 1 is lower than expected based on K_m_ differences for low loading rates. As loading rate increases first, sink 1 approaches saturation, enabling it to profit from its higher affinity. As loading rates increase further (beyond 10 nmol/s), sink 2 also approaches saturation, removing sink 1’s higher affinity benefit and causing partitioning to approach the 50% defined by the equal v_max_ values (Figure 4A, dashed line).

As above for the different unloading rates, source concentrations, source-sink concentration gradients, resistance ratio (Figure 4D), and between sink concentration, differences are larger in the biophysical model, explaining again the decreased advantage of sink 1 in the biophysical model (Figure 4A, solid line).

### Pathway Resistance

**FIGURE 5:**
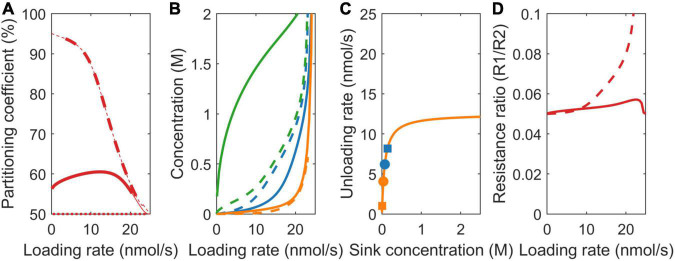
Impact of model choice on partitioning coefficients and the underlying mechanisms for different pathway resistance/length (r). **(A)** Partitioning coefficient for the VK (dotted), VKR (dashed), and biophysical (solid) models. The bold lines in **(A)** represent physiologically relevant conditions (0.1 < c_0_ < 2 M), showing that the biophysical model has a much broader physiologically relevant range. **(B)** Sink 1 (blue), sink 2 (orange), and source (green) sucrose concentrations. **(C)** The Michaelis–Menten curves for unloading rates, with dots representing the biophysical model and squares representing the VKR model. **(D)** Resistance ratios for the VKR (dashed) and biophysical (solid) models.

Sink organs are formed at different positions in the plant, with different transport pathway lengths from source to sink resulting in different pathway resistances, e.g., tubers form at a larger distance from the sucrose providing source leaves than young developing sink leaves. Importantly, resistance both affects and is dependent on concentration differences. For comparison purposes, we use equal baseline resistance values at zero sucrose concentrations for the two models, while allowing net, sucrose-dependent resistance values to differ. In the VK model, partitioning equals the v_max_ defined value of 50% (Figure 5A, dotted line), as all the sink parameters are equal and resistance is not incorporated.

For the VKR model, r_1_ << r_2_ (r_1_ = 7.5e12 Tmol s/m^6^ and r_2_ = 150 Tmol s/m^6^) implies that c_1_ >> c_2_ (Eq. 3), causing a reversal in the sink 1 and 2 concentration differences compared to the earlier two scenarios (Figure 5B, dashed lines). Additionally, between sink concentration, differences are significantly larger. The higher r_2_ now leads to lower sink 2 concentrations and, thus, a lower unloading rate in sink 2 when sinks are not yet saturated (Figure 5C, compare the position of blue vs. orange squares on the saturation curves), resulting in a higher partitioning toward sink 1 (Figure 5A, dashed line). When sink 1 becomes saturated (∼10 nmol/s), a further increase in loading rate increases unloading at sink 2, increasing c_2_, and decreasing sink 1 favored partitioning. As loading rate further increases, sink 2 also saturates and the v_max_ defined ratio of 50% partitioning is reached.

Again, in the biophysical model, source concentrations are higher, source-sink gradients are steeper (Figure 5B), and relative resistance differences are smaller (Figure 5D, r_1_/r_2_ slightly closer to 1 for unsaturated sinks). In the v_max_ and K_m_ scenarios, resistance differences oppose the v_max_ and K_m_ driven sink concentration differences, which, in turn, limit v_max_ and K_m_ driven resource partitioning advantages. Here, it is solely the resistance difference that drives concentration differences instead, with these concentration differences causing rather than reducing resource partitioning advantages. Reduced relative resistance differences in the biophysical model now reduce rather than enhance between sink concentration (Figure 5B) and saturation differences (Figure 5C, compare location of the dots representing the biophysical model with the squares representing the VKR model) and, thereby, limit the advantage of a lower resistance for sink 1 relative to the VKR model. Thus, for a broad range of loading rates, the disadvantage that a tuber experiences from its larger transport pathway length and resistance is significantly less than expected based on resistance differences alone.

### Xylem Water Flow Differences Affect Sucrose Partitioning

**FIGURE 6:**
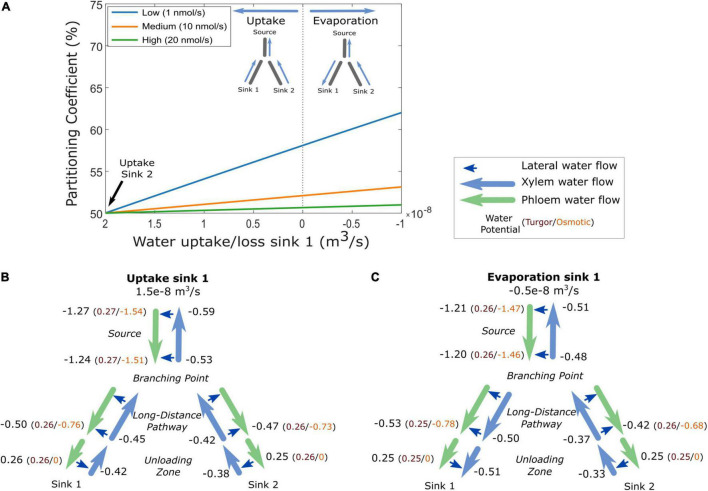
The effect of xylem boundaries on carbon partitioning between two sinks. **(A)** Partitioning coefficient for three loading rates as a function of xylem water boundary conditions in sink 1, for a constant sink 2 water uptake of 2e–8 m^3^/s. Water potentials of phloem and xylem (in MPa) at various locations in the model for a situation in which sink 1 evaporates at a rate of 0.5e–8 m^3^/s **(B)** or takes up water at a rate of 1.5e–8 m^3^/s **(C)** The scenarios in B and C are taken from the low-loading rate conditions of A. For the phloem water potential, the separate contributions of turgor and osmotic potential are given in brackets.

As phloem and xylem are hydraulically connected, changes in xylem water potential affect phloem water and solute transport (Sevanto et al., 2011; Savage et al., 2016; Konrad et al., 2019). An interesting question, thus, is whether, in addition to differences in sink characteristics and pathway resistance/length, differences between sink organs in terms of xylem water flow affect resource partitioning. Note that to investigate this only, the biophysically detailed model is suitable. To investigate the impact of xylem flow on resource partitioning, we used constant, equal VKR characteristics, a constant water uptake of sink 2 representing a root organ, while varying water flow in sink 1. When sink 1 xylem water uptake rate equals that of sink 2 (2e-8 m^3^/s), sucrose partitioning equals 50% (Figure 6A). A linear increase in sink 1 resource allocation occurred when moving from gradually decreasing water uptake to gradually increasing water evaporation in sink 1. This increased allocation to sink 1 is stronger for lower loading rates, when sinks are less saturated (Figure 6A). These results indicate that a smaller xylem counterflow and even more so a xylem co-flow increased partitioning toward a sink.

### Increased Turgor Gradient and Sucrose Concentration Causes Increased Sucrose Partitioning

To understand this phenomenon, we investigated scenarios in which sink 1 takes up water at a lower rate than sink 2 (Figure 6B) or evaporates water (Figure 6C). Both the lower water uptake and evaporation resulted in a more negative xylem potential in the pathway. Additionally, for an evaporating sink 1, a reversal in the xylem potential gradient occurs. As radial water flow is directed from high (least negative) to low (most negative) water potentials (Eq. 5b), differences in xylem potential impact lateral water flow and, thereby, phloem water potential. For phloem to deliver solutes to the sinks, water potentials in phloem and xylem must fulfill the constraint that lateral waterflow is directed toward the phloem in the source and long-distance pathway and toward the xylem in the sinks (Hölttä et al., 2006, 2009; Windt et al., 2006). The decreased (more negative) xylem water potential in the pathway toward sink 1, thus, dictates an accompanying decrease in phloem water potential that is largest in the scenario where sink 1 evaporates water. Phloem water potential consists of a turgor pressure (P) and osmotic potential (Π) component. Our simulations show that the decreased (more negative) phloem potential in the pathway toward sink 1 arises from a combination of lower turgor pressure and more negative osmotic potential in both the scenarios. A lower turgor pressure toward sink 1, combined with an equal turgor pressure at the branching point, results in a larger turgor pressure gradient and, hence, water flow toward sink 1 relative to sink 2. Additionally, the decreased osmotic potential arises from increased sucrose concentration in the pathway toward sink 1. The combined larger flow and higher sucrose concentration explain the increased sucrose partitioning toward sink 1, with a larger effect in the evaporation scenario where xylem and phloem potential become more negative.

### Resource Allocation Differences Between Roots, Tubers, and Developing Leaves

**FIGURE 7:**
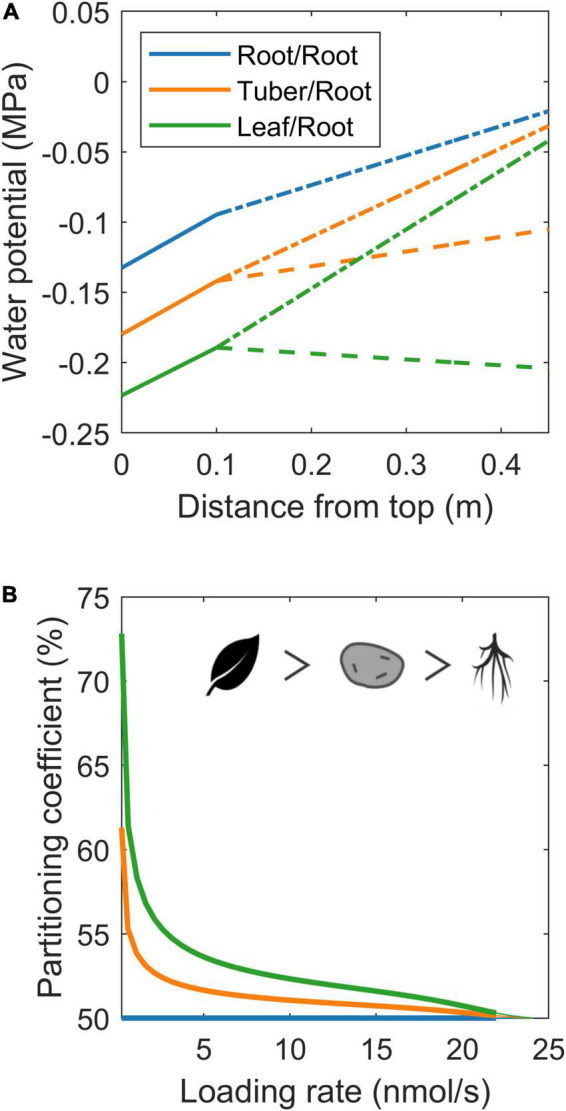
Resource allocation differences between roots, tubers, and developing leaves. **(A)** Xylem water potential along the plant for the three specific organ scenarios. Solid lines represent the water potential in the part of the plant stem containing source leaves, dashed lines represent the water potential along the long-distance pathway leading toward sink 1 (root/tuber/leaf), and dash-dotted lines represent the water potential along the long-distance pathway ending at sink 2 (root). **(B)** Partitioning coefficient as a function of loading rate for the same three organ type cases as shown in **(A)**. The symbols show the organ priority.

In the simulations earlier, we imposed water flow boundaries (influx or efflux rate) to systematically investigate the influence of xylem water flow rate and direction on resource partitioning. However, in most model applications, xylem water flow rate and direction are not a control parameter, but rather an emergent property from soil and atmosphere water potential or organ evaporation rates. We, therefore, also investigated 3 biologically realistic 2 sink scenarios (root-root, tuber-root, and young sink leaf-root), where we imposed a soil water potential of 0 MPa, simulating tubers by decreasing water uptake permeability by 90%, and young leaves through an evaporation rate of 10% of that of mature leaves. While this results in different, biologically more realistic, xylem water potentials (Figure 7A; Bland and Tanner, 1986), we again observe enhanced resource allocation to sinks with a reduced xylem counterflow (tuber vs. root) and even more so for xylem co-flow (leaf vs. root) (Figure 7B). Summarizing, we demonstrate that not only sink VK or pathway resistance properties affect resource partitioning, but that also differences in xylem water flow influence partitioning *via* the hydraulic connection with the phloem. Differences in xylem water flow cause tubers to have a resource partitioning advantage relative to the roots yet a disadvantage relative to sink leaves.

### Sucrose Efflux Further Aggravates Disadvantage of Roots and Tubers

Besides simulating the effect of xylem flow and radial water exchange between phloem and xylem, the biophysically detailed model is also capable of simulating the effects of radial sucrose exchange between phloem and the apoplast. This exchange is mediated by bidirectional SWEET transporters (Chen et al., 2012) and active SUC/SUT importers (Hafke et al., 2005). It was previously shown that sucrose efflux, retrieval, and efflux mitigation can strongly affect phloem transport characteristics and sucrose delivery to sinks (Minchin and Lacointe, 2017; van den Herik et al., 2021).

**FIGURE 8:**
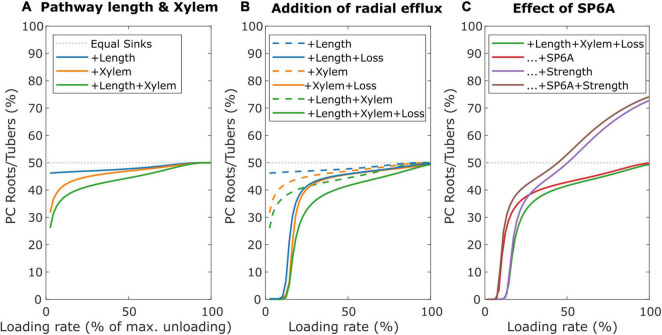
Individual and combined effects of pathway properties and SP6A on sucrose partitioning toward roots/tubers. **(A)** Individual and combined effect of pathway length and xylem water flow for further equal sink leaves and roots/tubers sink and pathway characteristics. **(B)** The effect of radial efflux on resource partitioning toward roots/tubers. **(C)** The dual effect of SP6A, mitigating efflux and increasing roots/tubers sink strength.

To investigate the impact of long-distance sucrose efflux, we simulate a young leaf and root/tuber, with initially equal sink and pathway properties (v_max_ = 5 nmol/s, K_m_ = 75 mM, l = 0.25 m), incrementally adding different properties affecting resource partitioning. First, we incorporated differences in source-sink distance, with the leaf sink located at 0.1 m and sink roots/tubers located at 0.3 m from the source, replicating a potato plant architecture. Like before, the sink with smaller pathway length, and, thus, resistance, experiences higher resource partitioning (Figure 8A, blue line). Second, we incorporated differences in xylem flow, setting leaf sink evaporation at 10% of that of mature leaves, and water potential in the soil at 0 MPa. Differences in xylem flow strongly benefit leaves sucrose partitioning (Figure 8A, orange line). Combining length and xylem flow differences demonstrate that these effects are largely additive (Figure 8A, green line).

Next, we incorporated SWEET-mediated sucrose efflux along the long-distance pathway, following a study by van den Herik et al. (2021), with radial efflux amounting to up to 23% of total sucrose loading rate. Trivially, sucrose export along the long-distance phloem aggravates the resource partitioning disadvantage due to an increased pathway length (Figure 8B, blue lines). However, also the disadvantage due to different xylem flow conditions (Figure 8B, orange lines) and their combination (Figure 8B, Green lines) are aggravated, indicating that xylem flow differences also impact radial sucrose transport. Note that, for low loading rates (below 15% of maximum unloading rate), partitioning toward tubers is close to 0%. For these low loading rates, while the phloem localized SWEET still experiences sufficient sucrose, the resulting efflux of sucrose along the pathway causes almost no sucrose to arrive at sinks. The longer pathway length and different xylem flow conditions result in larger sucrose efflux toward tubers, resulting in a near zero partitioning coefficient despite leaf sucrose yield also being very low (Supplementary Figure 3). Smaller length differences or lower leaf evaporation rate decreased the regime in which partitioning toward roots/tubers is 0% (Supplementary Figure 4).

### Efflux Mitigation and Increased Sink Strength Mediated by SP6A Work in Different Saturation Regimes

SWEET-mediated sucrose efflux is reduced by 40% upon the introduction of the FT-like protein SP6A in the phloem (Abelenda et al., 2019). However, there is currently no mechanism known that would restrict the loading and transport of SP6A specifically to tuber-directed phloem. We hypothesized that instead, the cumulative disadvantage tubers experience from their longer source-sink distance, xylem flow and sucrose export may enable undirected SP6A (i.e., present in all long-distance phloem) to preferentially increase tuber sucrose partitioning. Based on the experimental data, the undirected SP6A effect on sucrose transport was incorporated into our model by reducing for all the long-distance pathways, independent of the sink organ they are directed toward, the v_max_ parameter to 60% of its original value. Indeed, undirected SP6A introduction preferentially enhanced sucrose delivery toward roots/tubers (Figure 8C, red line) mostly through decreasing the regime of loading rates for which no sucrose reaches the roots/tubers due to radial efflux. Still, this SP6A effect does not enable tubers to become the dominant sucrose sinks. Besides its role in sucrose efflux mitigation, SP6A has been previously identified as important factor initiating tuberization (Navarro et al., 2011). During tuberization, the switch in unloading mode together with sink expansion increases sink strength. We, therefore, investigated the effect of an increased sink strength and the interplay with SP6A-mediated efflux mitigation. Experimental observations suggest sink-source feedback, with sink-strength affecting loading rates, likely through phloem sucrose levels (Chiou and Bush, 1998). We, therefore, assumed the tuber sink strength increase to be accompanied by an equal sized source strength increase. An increased tuber sink strength (5–15 nmol/s) enhanced tuber partitioning particularly at higher loading rates (Figure 8C, purple line). For very low loading rates, no sucrose arrived at the roots/tubers, rendering enhanced sink strength irrelevant. Beyond these loading rates, the effect of tuber sink strength increased with loading rate due to enhanced sink saturation (Supplementary Figure 5). As a result, for high-loading rates, tubers can now become the dominant sink organ. When combining SP6A-mediated efflux mitigation and sink strength increase (Figure 8C, brown line), we observe mostly additive effects, with efflux mitigation effects dominating at low and an increased sink strength effects dominating at high-loading rates. Combined, these results indicate that undirected SP6A sucrose efflux reduction broadens the range of loading rates over which tubers obtain significant amounts of sugars.

## Discussion

Yield of harvestable plant organs critically depends on sucrose partitioning between competing sinks. Understanding the mechanisms determining resource partitioning is, thus, of great agroeconomic relevance and modeling studies play an important role in unraveling the complex underlying processes. In this modeling study, we performed a systematic investigation into the individual and combined effects of sink characteristics (unloading strength, sucrose affinity, and water uptake or evaporation) and pathway properties (length, resistance, and radial sucrose efflux) on sucrose partitioning, taking potato tuber sucrose delivery as an example case.

We demonstrated that in our biophysically detailed model, the effects of sink strength and affinity, as well as pathway resistance/length, are significantly enhanced compared to earlier studies using simplified sucrose transport models (Minchin et al., 1993; Bancal and Soltani, 2002). These differences could be attributed to the underestimation of sucrose concentration and pathway resistance in the simplified models. We further show that these effects are relevant in a much broader loading rate range compared to a study by Bancal and Soltani (2002) due to higher source concentrations. Our findings on the importance of pathway properties are supported by recent findings that source-sink distance differences drive divergence in yield between grapes (Pallas et al., 2010; Zhu et al., 2021). Additionally, we observed that the previously reported phenomenon of weakest sink prioritization—the larger than expected sucrose allocation to the weaker sink for non-saturating sucrose loading—occurs not only for sinks differing in sink strength, but also for differences in sucrose affinity or pathway length.

Interestingly, we found that in addition to phloem pathway length, the rate and direction of the coupled, parallel xylem flow also impacted sink partitioning. For equal sink characteristics, sucrose partitioning to a sink linearly increased with xylem flow and potential. This effect could be explained from the hydraulic coupling between phloem and xylem and the concomitant changes in both the phloem osmotic and turgor pressure. Thus, while previous study focused on the impact of xylem flow *via* leaf water potential and photosynthetic activity on plant organ growth (Solari et al., 2006), we show that even for constant photosynthesis and, hence, loading rates, the hydraulic coupling between xylem and phloem causes xylem flow to impact sucrose transport and growth.

In the case of potato tubers, we showed that their limited water uptake causes them to be at an advantage in terms of resource partitioning relative to roots that take-up considerably more water, yet at a disadvantage to young leaves that evaporate limited amounts of water. An additional disadvantage arises from the larger distance tubers and roots that have compared to young leaves from source leaves. Both the disadvantages are increased when including SWEET-mediated radial sucrose export. This exacerbated disadvantage enables undirected SP6A-mediated export mitigation to preferentially benefit root/tuber resource allocation. This export mitigating effect of SP6A that dominates at lower loading rates for which phloem transport resistance and xylem water flow differences are relevant complements the effect of SP6A on tuber sink strength that becomes fully effective for high, saturating loading rates. Overall, the undirected SP6A signal significantly broadens the range of loading rates over which tubers obtain significant amounts of sugars.

A major point of discussion is the *in-planta* relevance of the reported results. In essence, this issue revolves around the question whether sinks under most physiological conditions operate at/near saturation or rather operate under non-saturating conditions. As our, and previous results (Minchin et al., 1993; Bancal and Soltani, 2002) show, under saturating conditions, the ratio between sink strength (v_max_) dictates resource partitioning. It is only under non-saturating conditions that other factors such as sink affinity, pathway resistance/length, xylem flow rate and direction, and radial sucrose efflux significantly affect resource partitioning, weakest sink prioritization occurs, and SP6A efflux mitigation effects weigh in. Classical experiments have demonstrated that upon chemically (Farrar and Minchin, 1991) or cooling induced sink strength reduction (Minchin et al., 1997), sucrose import in unaffected sinks adjusts to the new conditions on a timescale of hours. This slow adaptation has been taken as evidence for sink saturation, reasoning that enhanced uptake requires upregulation of sink uptake capacity (Minchin and Lacointe, 2005). Here, we challenge this view. First, only if remaining sinks are unable to import *any* additional sucrose would this support prior sink saturation, while if remaining sinks are unable to import *all* the extra sucrose immediately this merely implies that maximum sink uptake capacity is now exceeded. Second, upon enhanced sucrose availability, sucrose metabolism requires upregulation. This delayed increase in utilization could lead to sucrose accumulation with negative feedback on sink uptake limiting the initial increase in uptake (Farrar and Minchin, 1991). Similarly, feedback regulation between sink demand and source supply may result in reduced source loading upon sink removal due to sugar accumulation in phloem or leaves, limiting actual extra sucrose availability. Finally, our model generates a 1–2 h timescale for partitioning to adapt to instant changes in one sink, without any changes occurring in the second sink (Supplementary Figure 6). This demonstrates that delayed adaptation of partitioning can at least partly be explained by slow adaptation of the long-distance transport and does not necessarily imply sink upregulation and prior saturation.

In further support of the relevance of pathway properties and incomplete sink saturation for resource partitioning, many stress conditions such as water shortage, salt stress, and infections will reduce harvestable organ sucrose availability through either reducing photosynthesis efficiency or affecting energy budget allocation. We, thus, stress that while simple partitioning models, such as relative growth rate approaches, may serve as a first-order approximation for predicting harvestable organ yields, they do not take into account all the relevant factors. Therefore, in case, substantial deviations with experimental observations occur or more detailed predictions are need, more biophysically detailed and biologically realistic models are essential. These are particularly relevant when studying regulatory mechanisms impinging on sucrose transport, such as the SP6A-SWEET interaction studied here. For future study, it would be of great interest to study resource partitioning in a dynamic, growing architecture. Here, we used a static architecture to study the effects of sink and pathway properties, as inclusion of a growing, more complex, architecture would generate complex feedbacks, further complicating interpretation of the results. Similarly, this model does not include variations in sieve element architecture or vascular bundle numbers along the long-distance phloem or between different organ types. Including such details in the hydraulic architecture of the model are expected to further improve our understanding and capability to predict resource partitioning. Nonetheless, with the insights and modeling from this article, it should now already be possible to interpret resource partitioning in more complex scenarios, which can give insights in important agroeconomic factors such as tuber size distribution.

## Data Availability Statement

The original contributions presented in the study are included in the article/Supplementary Material, further inquiries can be directed to the corresponding author/s.

## Author Contributions

BH developed, implemented, analyzed the model, and wrote the manuscript. KT conceived the project, analyzed the model, and wrote the manuscript. Both authors contributed to the article and approved the submitted version.

## Conflict of Interest

The authors declare that the research was conducted in the absence of any commercial or financial relationships that could be construed as a potential conflict of interest.

## Publisher’s Note

All claims expressed in this article are solely those of the authors and do not necessarily represent those of their affiliated organizations, or those of the publisher, the editors and the reviewers. Any product that may be evaluated in this article, or claim that may be made by its manufacturer, is not guaranteed or endorsed by the publisher.

## References

[B1] Abelenda J. A. Bergonzi S. Oortwijn M. Sonnewald S. Du M. Visser R. G. F. (2019). Source-Sink Regulation Is Mediated by Interaction of an FT Homolog with a SWEET Protein in Potato. *Curr. Biol*. 29 1178–1186. 10.1016/j.cub.2019.02.018 30905604

[B2] Bancal P. Soltani F. (2002). Source–sink partitioning. Do we need Münch? *J. Exp. Bot.* 53 1919–1928. 10.1093/jxb/erf037 12177131

[B3] Bland W. L. Tanner C. B. (1986). Potato tuber water potential components during storage. *Am. Potato J.* 63 649–653. 10.1007/BF02852927

[B4] Braun D. M. Wang L. Ruan Y.-L. (2014). Understanding and manipulating sucrose phloem loading, unloading, metabolism, and signalling to enhance crop yield and food security. *J. Exp. Bot.* 65 1713–1735. 10.1093/jxb/ert416 24347463

[B5] Chen L.-Q. Qu X.-Q. Hou B.-H. Sosso D. Osorio S. Fernie A. R. (2012). Sucrose Efflux Mediated by SWEET Proteins as a Key Step for Phloem Transport. *Science* 335 207–211. 10.1126/science.1213351 22157085

[B6] Chiou T.-J. Bush D. R. (1998). Sucrose is a signal molecule in assimilate partitioning. *Proc. Natl. Acad. Sci. U. S. A.* 95 4784–4788. 10.1073/pnas.95.8.4784 9539816 PMC22568

[B7] Clerx L. E. Rockwell F. E. Savage J. A. Holbrook N. M. (2020). Ontogenetic scaling of phloem sieve tube anatomy and hydraulic resistance with tree height in Quercus rubra. *Am. J. Bot.* 107 852–863. 10.1002/ajb2.1481 32468597

[B8] Da Silva D. Qin L. DeBuse C. DeJong T. M. (2014). Measuring and modelling seasonal patterns of carbohydrate storage and mobilization in the trunks and root crowns of peach trees. *Ann. Bot.* 114 643–652. 10.1093/aob/mcu033 24674986 PMC4156119

[B9] de Vries J. Evers J. B. Kuyper T. W. Ruijven J. Mommer L. (2021). Mycorrhizal associations change root functionality: a 3D modelling study on competitive interactions between plants for light and nutrients. *New Phytol.* 231 1171–1182. 10.1111/nph.17435 33930184 PMC8361744

[B10] de Wit A. Boogaard H. Fumagalli D. Janssen S. Knapen R. van Kraalingen D. (2019). 25 years of the WOFOST cropping systems model. *Agric. Syst.* 168 154–167. 10.1016/j.agsy.2018.06.018

[B11] Driever S. M. Simkin A. J. Alotaibi S. Fisk S. J. Madgwick P. J. Sparks C. A. (2017). Increased SBPase activity improves photosynthesis and grain yield in wheat grown in greenhouse conditions. *Philos. Trans. R. Soc. Lond. B Biol. Sci.* 372 1–10. 10.1098/rstb.2016.0384 28808101 PMC5566882

[B12] Farrar J. F. Minchin P. E. H. (1991). Carbon Partitioning in Split Root Systems of Barley: relation to Metabolism. *J. Exp. Bot.* 42 1261–1269. 10.1093/jxb/42.10.1261 12432039

[B13] Fernie A. R. Bachem C. W. B. Helariutta Y. Neuhaus H. E. Prat S. Ruan Y.-L. (2020). Synchronization of developmental, molecular and metabolic aspects of source–sink interactions. *Nat. Plants* 6 55–66. 10.1038/s41477-020-0590-x 32042154

[B14] Hafke J. B. Amerongen J. K. van, Kelling F. Furch A. C. U. Gaupels F. (2005). Thermodynamic Battle for Photosynthate Acquisition between Sieve Tubes and Adjoining Parenchyma in Transport Phloem. *Plant Physiol.* 138 1527–1537. 10.1104/pp.104.058511 15980202 PMC1176423

[B15] Hölttä T. Mencuccini M. Nikinmaa E. (2009). Linking phloem function to structure: analysis with a coupled xylem–phloem transport model. *J. Theor. Biol.* 259 325–337. 10.1016/j.jtbi.2009.03.039 19361530

[B16] Hölttä T. Vesala T. Sevanto S. Perämäki M. Nikinmaa E. (2006). Modeling xylem and phloem water flows in trees according to cohesion theory and Münch hypothesis. *Trees* 20 67–78. 10.1007/s00468-005-0014-6

[B17] Konrad W. Katul G. Roth-Nebelsick A. Jensen K. H. (2019). Xylem functioning, dysfunction and repair: a physical perspective and implications for phloem transport. *Tree Physiol.* 39 243–261. 10.1093/treephys/tpy097 30299503

[B18] Lacointe A. Minchin P. E. H. (2008). Modelling phloem and xylem transport within a complex architecture. *Funct. Plant Biol.* 35:772. 10.1071/FP08085 32688831

[B19] Lescourret F. Moitrier N. Valsesia P. Génard M. (2011). QualiTree, a virtual fruit tree to study the management of fruit quality. I. Model development. *Trees* 25 519–530. 10.1007/s00468-010-0531-9

[B20] Minchin P. E. H. Lacointe A. (2005). New understanding on phloem physiology and possible consequences for modelling long-distance carbon transport. *New Phytol.* 166 771–779. 10.1111/j.1469-8137.2005.01323.x 15869640

[B21] Minchin P. E. H. Lacointe A. (2017). Consequences of phloem pathway unloading/reloading on equilibrium flows between source and sink: a modelling approach. *Funct. Plant Biol.* 44:507. 10.1071/FP16354 32480583

[B22] Minchin P. E. H. Thorpe M. R. Farrar J. F. (1993). A Simple Mechanistic Model of Phloem Transport which Explains Sink Priority. *J. Exp. Bot.* 44 947–955. 10.1093/jxb/44.5.947 12432039

[B23] Minchin P. E. H. Thorpe M. R. Wünsche J. N. Palmer J. W. Picton R. F. (1997). Carbon partitioning between apple fruits: short- and long-term response to availability of photosynthate. *J. Exp. Bot.* 48 1401–1406. 10.1093/jxb/48.7.1401 12432039

[B24] Morison K. R. (2002). “Viscosity equations for sucrose solutions: old and new 2002,” *Proceedings of the 9th APCChE Congress and CHEMECA*.

[B25] Navarro C. Abelenda J. A. Cruz-Oró E. Cuéllar C. A. Tamaki S. Silva J. (2011). Control of flowering and storage organ formation in potato by FLOWERING LOCUS T. *Nature* 478 119–122. 10.1038/nature10431 21947007

[B26] Nölke G. Houdelet M. Kreuzaler F. Peterhänsel C. Schillberg S. (2014). The expression of a recombinant glycolate dehydrogenase polyprotein in potato (*Solanum tuberosum*) plastids strongly enhances photosynthesis and tuber yield. *Plant Biotechnol. J.* 12 734–742. 10.1111/pbi.12178 24605946

[B27] Pallas B. Christophe A. Lecoeur J. (2010). Are the common assimilate pool and trophic relationships appropriate for dealing with the observed plasticity of grapevine development? *Ann. Bot.* 105 233–247. 10.1093/aob/mcp278 19946042 PMC2814752

[B28] Perämäki M. Nikinmaa E. Sevanto S. Ilvesniemi H. Siivola E. Hari P. (2001). Tree stem diameter variations and transpiration in Scots pine: an analysis using a dynamic sap flow model. *Tree Physiol.* 21 889–897. 10.1093/treephys/21.12-13.889 11498336

[B29] Savage J. A. Clearwater M. J. Haines D. F. Klein T. Mencuccini M. Sevanto S. (2016). Allocation, stress tolerance and carbon transport in plants: how does phloem physiology affect plant ecology? *Plant Cell Environ.* 39 709–725. 10.1111/pce.12602 26147312

[B30] Schulze W. Weise A. Frommer W. B. Ward J. M. (2000). Function of the cytosolic N-terminus of sucrose transporter AtSUT2 in substrate affnity. *FEBS Lett.* 485 189–194. 10.1016/s0014-5793(00)02180-3 11094165

[B31] Sevanto S. Hölttä T. Holbrook N. M. (2011). Effects of the hydraulic coupling between xylem and phloem on diurnal phloem diameter variation. *Plant Cell Environ.* 34 690–703. 10.1111/j.1365-3040.2011.02275.x 21241327

[B32] Solari L. I. Johnson S. DeJong T. M. (2006). Relationship of water status to vegetative growth and leaf gas exchange of peach (Prunus persica) trees on different rootstocks. *Tree Physiol.* 26 1333–1341. 10.1093/treephys/26.10.1333 16815835

[B33] Thompson M. V. Holbrook N. M. (2003). Application of a Single-solute Non-steady-state Phloem Model to the Study of Long-distance Assimilate Transport. *J. Theor. Biol.* 220 419–455. 10.1006/jtbi.2003.3115 12623280

[B34] van den Herik B. Bergonzi S. Bachem C. W. B. Tusscher K. (2021). Modelling the physiological relevance of sucrose export repression by an Flowering Time homolog in the long-distance phloem of potato. *Plant Cell Environ.* 44 792–806. 10.1111/pce.13977 33314152 PMC7986384

[B35] Viola R. Roberts A. G. Haupt S. Gazzani S. Hancock R. D. Marmiroli N. (2001). Tuberization in Potato Involves a Switch from Apoplastic to Symplastic Phloem Unloading. *Plant Cell* 13 385–398. 10.1105/tpc.13.2.385 11226192 PMC102249

[B36] Windt C. W. Vergeldt F. J. Jager P. A. D. As H. V. (2006). MRI of long-distance water transport: a comparison of the phloem and xylem flow characteristics and dynamics in poplar, castor bean, tomato and tobacco. *Plant Cell Environ.* 29 1715–1729. 10.1111/j.1365-3040.2006.01544.x 16913861

[B37] Zhu J. Gou F. Rossouw G. Begum F. Henke M. Johnson E. (2021). Simulating organ biomass variability and carbohydrate distribution in perennial fruit crops: a comparison between the common assimilate pool and phloem carbohydrate transport models. *In Silico Plants* 3:diab024. 10.1093/insilicoplants/diab024

