# Supplementary material for: Undirected Sucrose Efflux Mitigation by the FT-Like SP6A Preferentially Enhances Tuber Resource Partitioning
Source: Front Plant Sci. 2022 May 9;13:817909. doi: 10.3389/fpls.2022.817909 (PMC9125184; doi:10.3389/fpls.2022.817909)
Supplement: Supplementary file 1 [file Data_Sheet_1.docx]

**Supplementary material for:**

**Undirected sucrose efflux-mitigation by the FT-like SP6A preferentially enhances tuber resource partitioning**

Bas van den Herik^1^, Kirsten ten Tusscher^1*^

1. Computational Developmental Biology, Utrecht University, Utrecht, The Netherlands

*Corresponding author: Kirsten ten Tusscher, [K.H.W.J.tenTusscher@uu.nl](mailto:K.H.W.J.tenTusscher@uu.nl), Computational Developmental Biology Group, Department of Biology, Faculty of Science, Utrecht University, Padualaan 8, 3584 CH Utrecht, the Netherlands, +31302533637


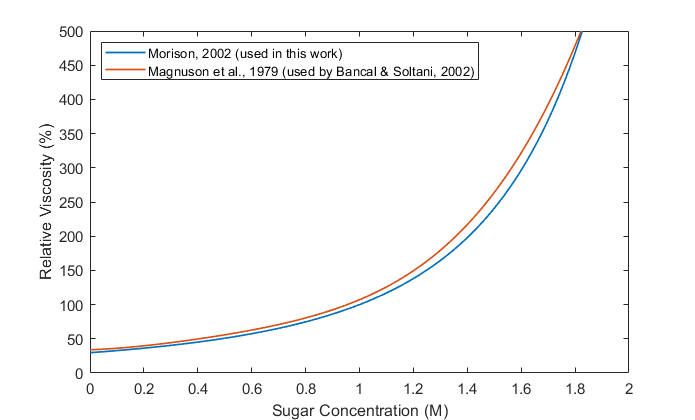


**Figure S1.** Relative viscosity (compared to a viscosity of 3MPa s for Magnuson et al, and the viscosity at 1M for Morison) as used in the VKR (red line) and biophysically detailed (blue line) models.


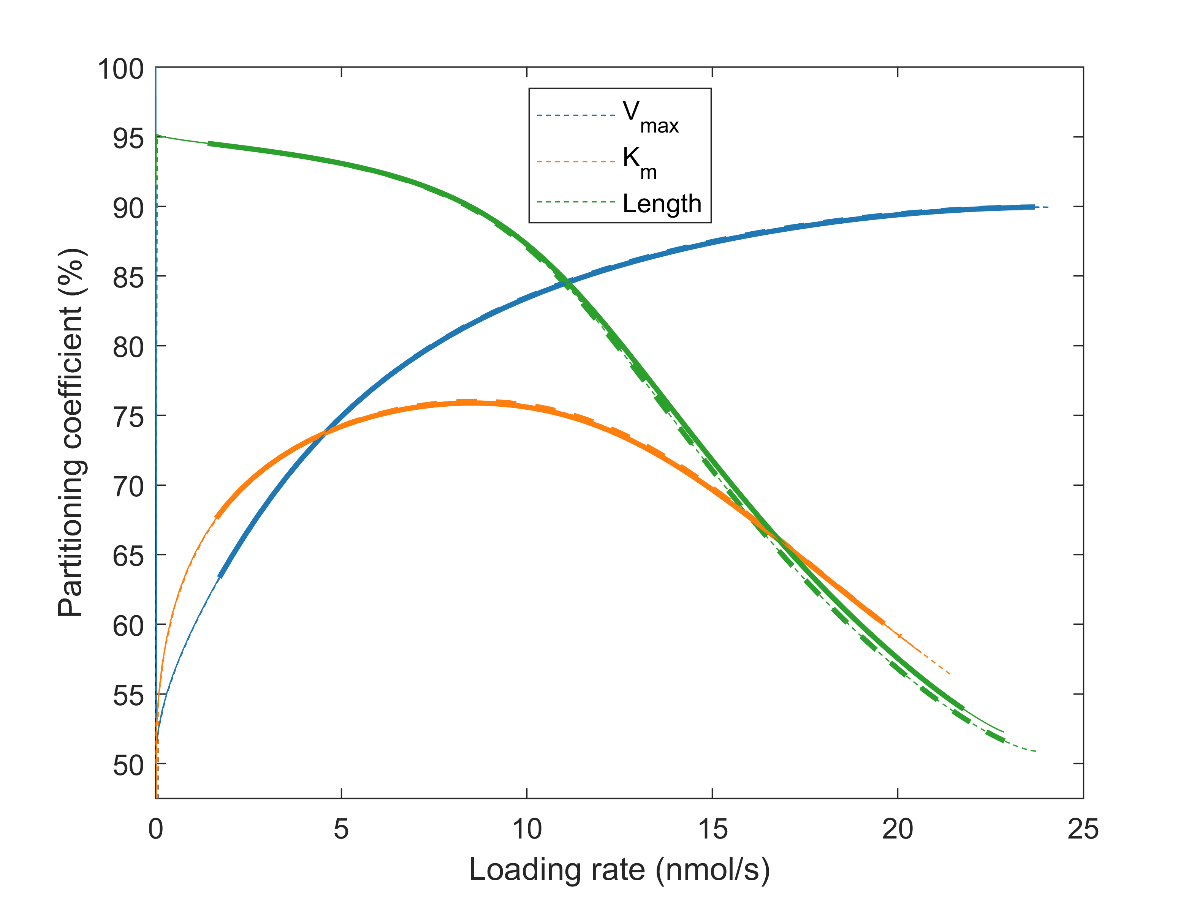


**Figure S2. Effect of a non-zero shared pathway resistance.** Bold lines depict a simulation of the biophysically detailed model without a shared pathway with standard sink characteristics. Dashed lines depict a simulation in which a shared pathway of 0.25m was included.


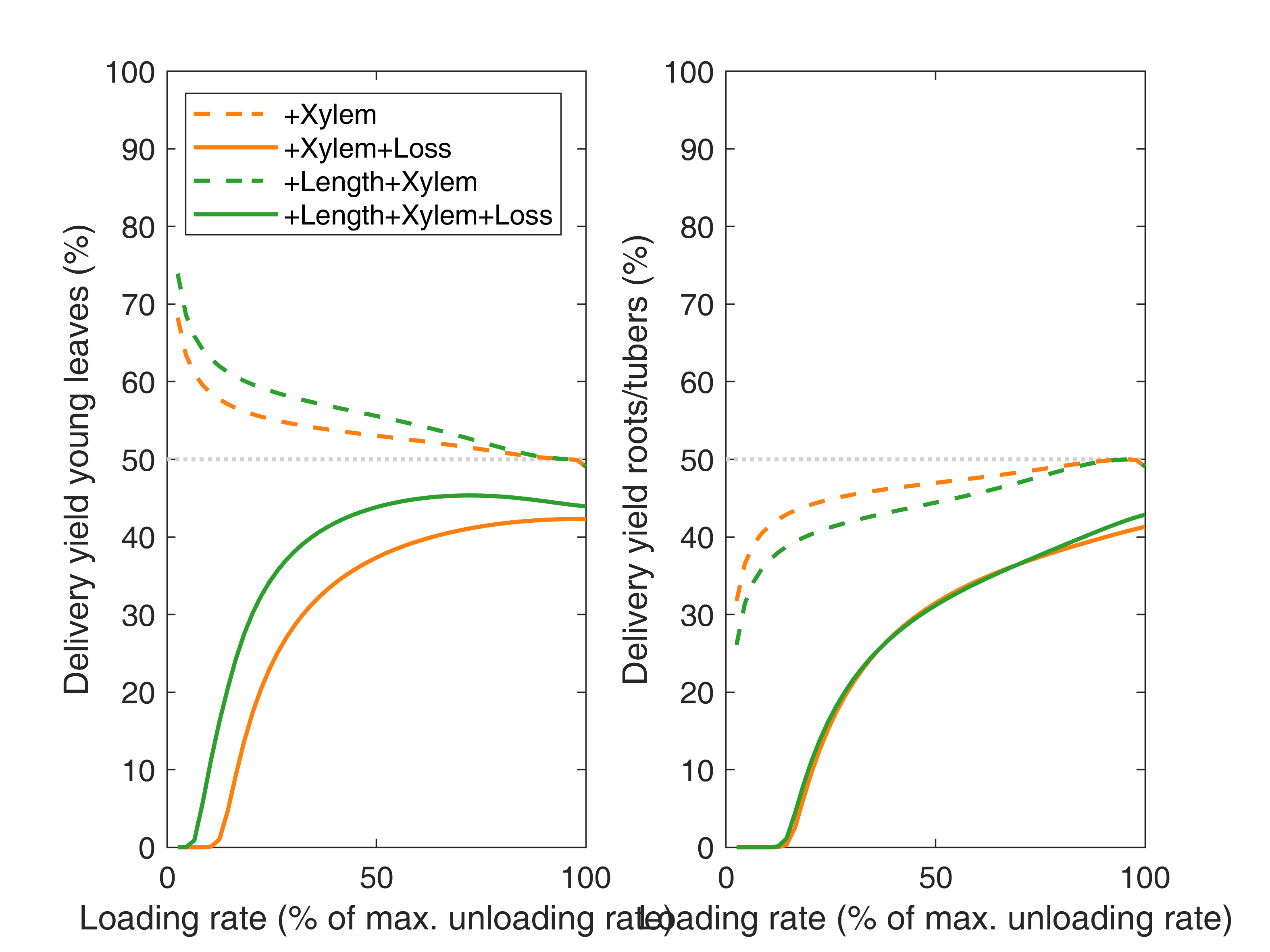
**Fig S3. Delivery yield (v_i_/v_o_)** for young leaves (A) and roots/tubers (B) for the same simulations as shown in Fig. 7B. Delivery yield is defined as sink unloading over total loading rate. For no radial efflux this is equal to the partitioning coefficient, but when including radial loss these two measures differ. Delivery yield specifically shows delivered sucrose to a sink, relative to total sucrose loading, instead of relative to total sucrose unloading at the sink. For the loss scenario, it can clearly be seen that for low loading rates no sucrose reaches the root/tuber sinks and almost non reaches the leaves, making that the partitioning coefficient gives an incomplete representation of the situation.


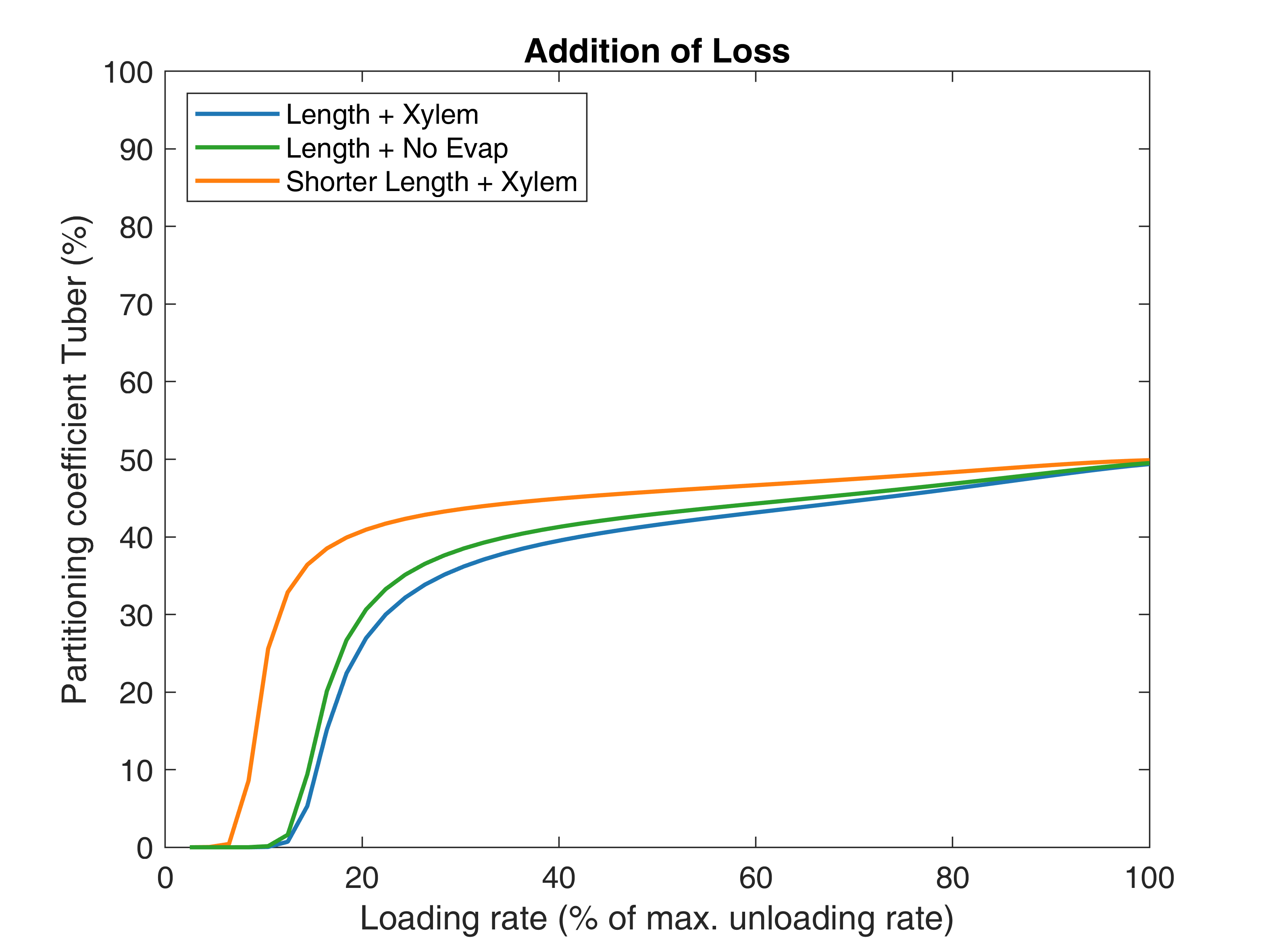
**Fig 4 Partitioning towards roots/tubers** for smaller pathway length differences (0.1m/0.15m instead of 0.1m/0.3m, orange line) and without evaporation in the leaf sinks (green line). The blue line shows conditions as used in fig. 7B, i.e., with 0.3m distance of roots/tubers and evaporation in the leaf sink.


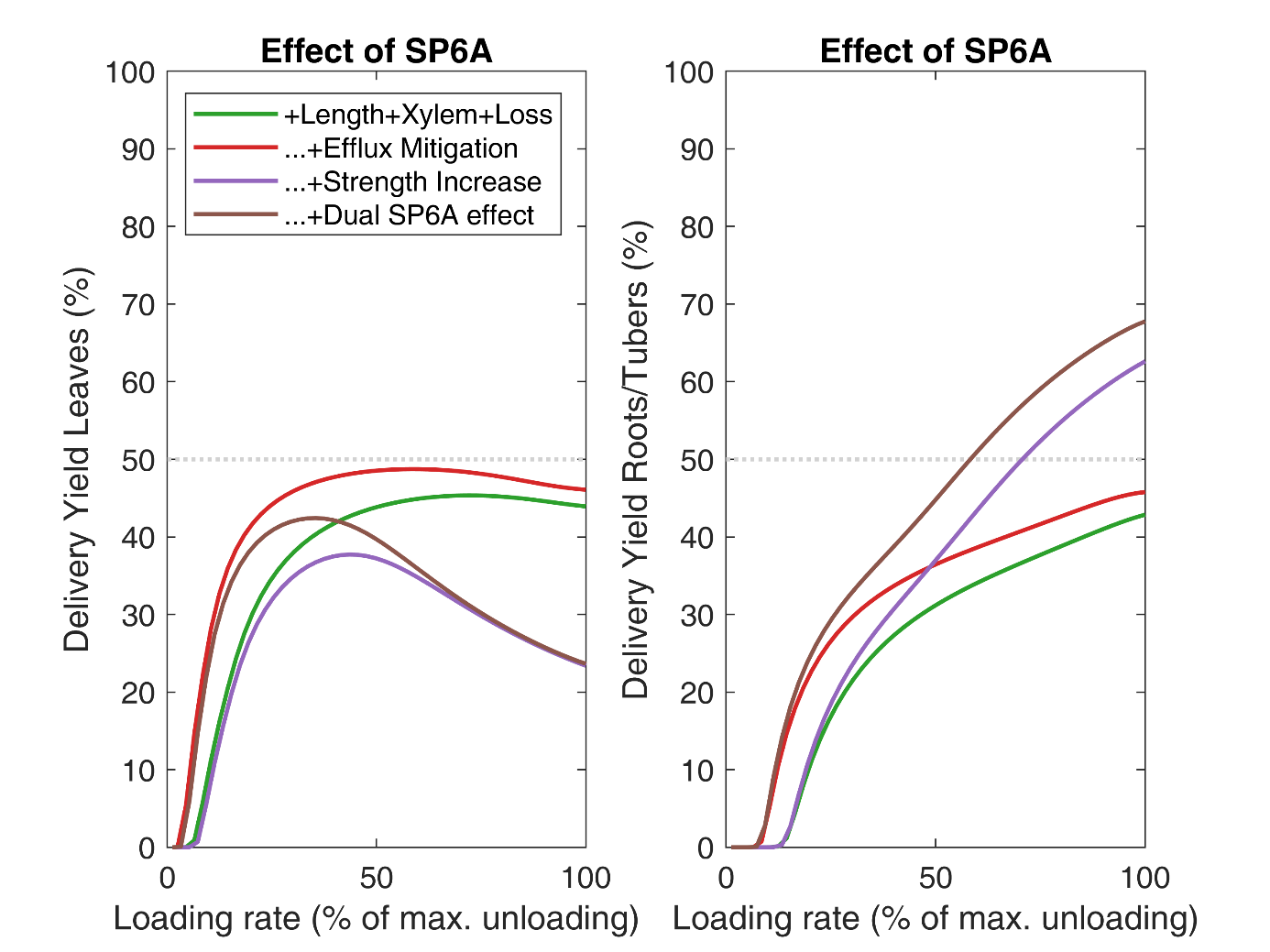
**Fig. S5.** **Delivery yield (v_i_/v_o_)** for young leaves (A) and roots/tubers (B) for the same simulations as shown in Fig. 7C. Increased sink-strength of tubers makes that not only the partitioning towards tubers increased, but also total sucrose delivery becomes dominant.


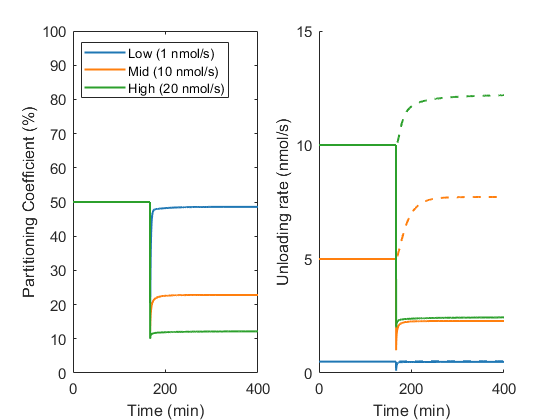


**Fig. S6. Time dynamics of the adaptation of partitioning to changed sink-conditions.** At t=180 min sink unloading rate was changed from equal for both sinks (12.5nmol/s) to 2.5nmol/s for sink 1 (solid lines) whereas sink 2 was kept equal at 12.5nmol/s (dashed line). Adaptation to this sink change took approximately 2 hours for high loading rates, and about 1 hour for low loading rates. For lower loading rates the adaptation time was shorter due to strong influence of the weakest sink prioritization.
